# Supplementary material for: Systemic and intrinsic functions of ATRX in glial cell fate and CNS myelination in male mice
Source: Nat Commun. 2023 Nov 4;14:7090. doi: 10.1038/s41467-023-42752-y (PMC10625541; doi:10.1038/s41467-023-42752-y)

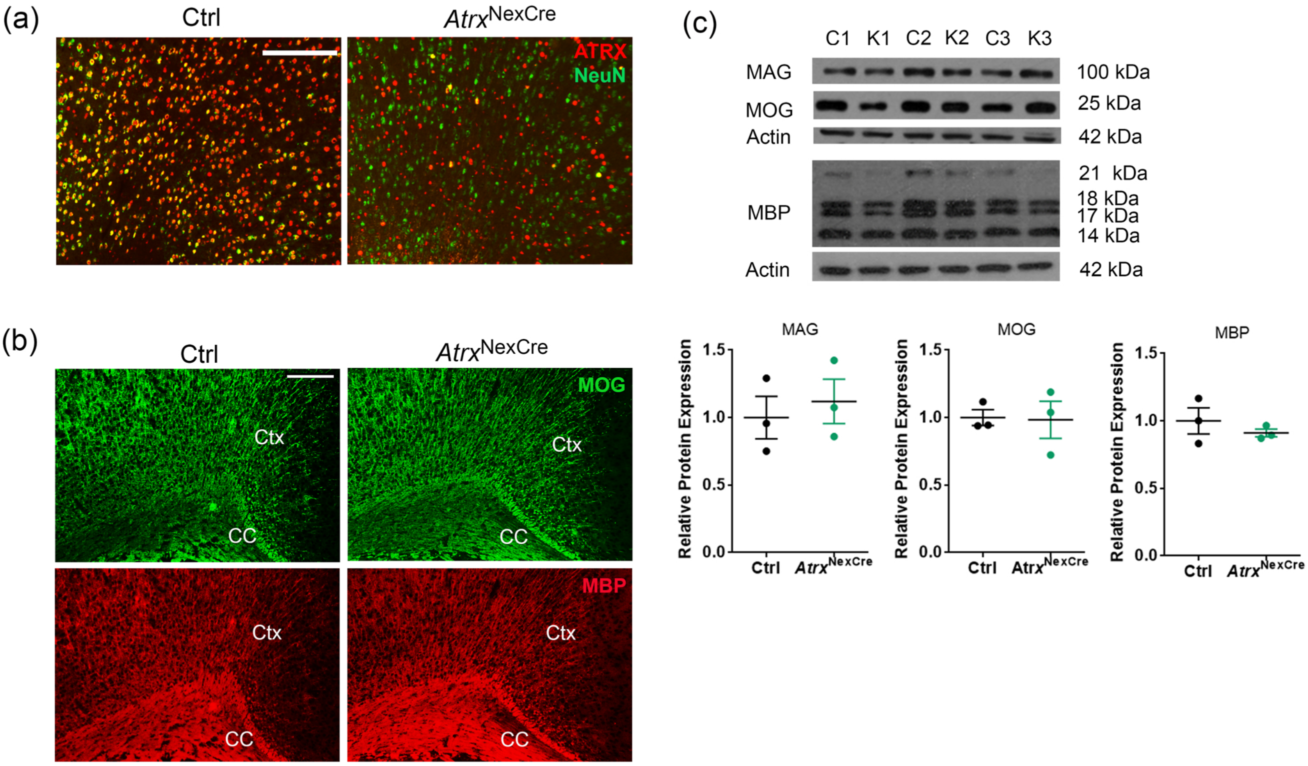

Figure S1: Normal myelination in mice with *Atrx* deletion in forebrain excitatory neurons. (a) Immunofluorescence staining of P20 brain cryosections shows absence of ATRX protein (red) in NeuN-expressing neurons (green) in the cortex of *Atrx*^{NexCre} mice. Scale bar, 100 µm. (b) MBP and MOG immunofluorescence staining of P20 *Atrx*^{NexCre} and control brain cryosections. Scale bar, 100 µm. (c) Western blot analysis of MAG, MOG and MBP and quantification below shows unaltered level of these proteins in the P20 *Atrx*^{NexCre} mouse forebrain compared to controls when normalized to beta-actin protein levels (MAG p=0.63, MOG p=0.92, MBP p=0.43; Two-sided Student's T-test). Data are presented as mean values +/- SEM, n=3 animals for each genotype.

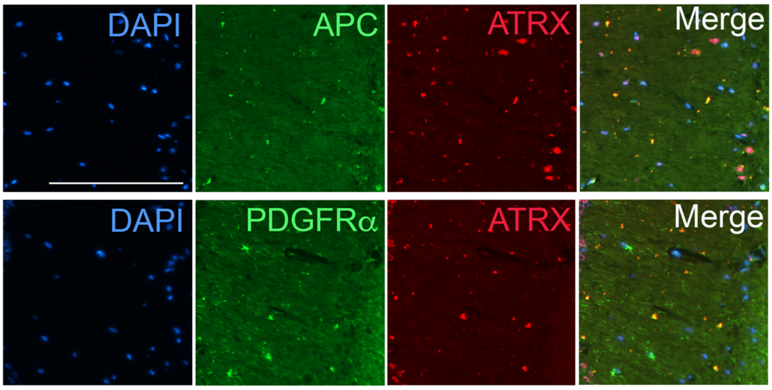

Figure S2. The ATRX protein is expressed in OPCs and OLs in the mouse brain. Immunofluorescence co-staining of the mouse hippocampus with anti-ATRX and anti-PDGFRα antibodies (OPC marker) or anti-ATRX and anti-APC (OL marker). Scale bar, 200μM.

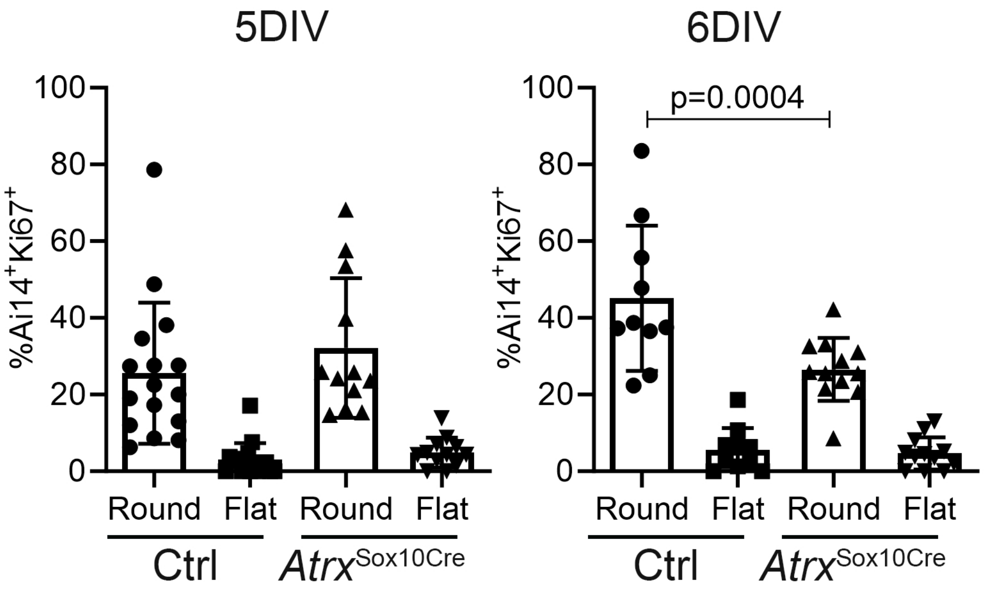

Figure S3. Proliferation of ATRX-null and control OPCs in mixed glial primary cultures. Ki67 staining reveals normal proliferation at 5DIV (Ctrl n=16 images scored for cultures established from 4 animals; *Atrx*^{Sox10Cre} n=12 images scored for cultures established from 4 animals). Decreased proliferation is observed for round-shaped ATRX-null cells at 6DIV ($p=0.0004$, Ctrl n=10 images scored for cultures established from 3 animals; *Atrx*^{Sox10Cre} n=12 images scored for 3 animals. In all experiments, >600 cells were scored for each genotype. Data are presented as mean values +/- SD analyzed by one-way ANOVA with post-hoc Tukey HSD.

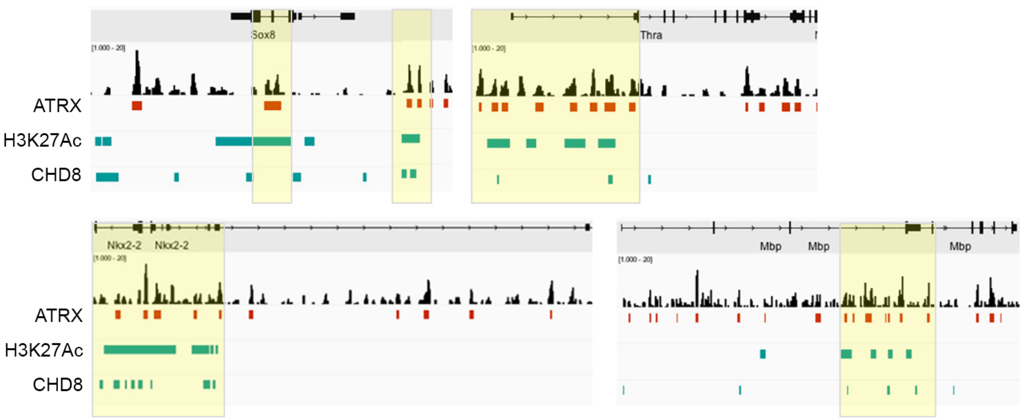

Figure S4. ChIP-seq of ATRX, H3K27Ac and CHD8 at oligodendrocyte lineage genes in OPCs. IGV views of genomic regions corresponding to *Sox8*, *Thra*, *Nkx2-2* and *Mbp* genes. ATRX ChIP-seq data is shown as sequence peaks in black and called peaks in red. H3K27Ac and CHD8 called peaks are shown in green. Yellow boxes highlight major regions of overlap.

Supplementary Table 1: List of primer sequences

Gene Name	Forward Primer	Reverse Primer	Application
AtrxWT	AGA AAT TGA GGA TGC TTC ACC	TGA ACC TGG GGA CTT CTT TG	Genotyping
Atrxfloxed	AGA AAT TGA GGA TGC TTC ACC	CCA CCA TGA TAT TCG GCA AG	Genotyping
FoxG1Cre	TGA CCA GAG TCA TCC TTA GCG	AAT GCT TCT GTC CGT TTG CC	Genotyping
NexCre	TGA CCA GAG TCA TCC TTA GCG	AAT GCT TCT GTC CGT TTG CC	Genotyping
Sox10Cre	CAC CTA GGG TCT GGC ATG T	CAG GTT TTG GTG CAC AGT CA	Genotyping
Sun1GFP	AAG GGA GCT GCA GTG GAG TA	CGG GCC ATT TAC CGT AAG TTA T	Genotyping
Ai14Tomato	GGC ATT AAA GCA GCG TAT CC	CTG TTC CTG TAC GGC ATG G	Genotyping
Gapdh	CAA CGA CCC CTT CAT TGA CCT	ATC CAC GAC CGA CAC ATT GG	qRT-PCR
Atrx	AGA AAT TGA GGA TGC TTC ACC	TGA ACC TGG GGA CTT CTT TG	qRT-PCR
Olig2	GCA GCG AGC ACC TCA AAT CT	AGA TCA TCG GGT TCT GGG GA	qRT-PCR
Sox10	CTA CAGG GAG TGC CCA CCT GG	GCT CTG TCT TTG GGG TGG TT	qRT-PCR
Pdgfrα	GTC AGG CCA CT AAA GAG GTC A	CGT GCA AGT TGA CAG CTT CC	qRT-PCR
Olig1	CTC GCC CAG GTG TTT TGT TG	CGA CGT GCC TTG CTA CCT AT	qRT-PCR
Gpr17	TCACAGCTTACCTGCTTCCC	TTGTCCGCATTGCTCAGAGT	qRT-PCR
Cspg4	CTCAAGATGGGAGCCTCAGC	ACAAAGGCGTCTGTCTGTGT	qRT-PCR
Mag	CAG AGA GCC ACT GCC TTC AA	TCA AAG GCC ACA GAG GTT C	qRT-PCR
Mog	AGA GGC AGC AAT GGA GTT GA	TGC GAT GAG AGT CAG CAC AC	qRT-PCR
Mbp	CAT TGG GTC GCC ATG GGA AA	AGC CTC TCC TCG GTG AAT CT	qRT-PCR
Plp	GGC CAC TGG ATT GTG TTT CT	GAA AGC ATT CCA TGG GAG AA	qRT-PCR
Gfap	AAA CCA GCC TGG ACA CCA AA	ACA CCT CAC ATC ACC ACG TC	qRT-PCR
S100β	TGA AGC CAG AGA GGA CTC CA	CCA GGA AGT GAG AGA GCT CG	qRT-PCR
Olig2A	ATT CCC CGT CTC ACT CCG TA	TAG CGG GGC TGC TAA AGA AG	ChIP qRT-PCR
Olig2B	GCC TGA CGC TAC AGT GAC AA	TAG CGG GGC TGC TAA AGA AG	ChIP qRT-PCR
Olig2C	CTG CCT CCA CCC AGC TA TAA	GGT GTT GGC TCG GTC TGT AA	ChIP qRT-PCR
Olig2D	ATC TTC CTC CAG CAC CTC CT	GTT CGC GGC TGT TGA TCT TC	ChIP qRT-PCR
Olig2E	TAG GAG ACT CCC AGG AAC CG	GAC CAC AGG ACC CTA AGT GC	ChIP qRT-PCR
Olig16kb	CCT CCC ACA CAC ACA CCT TT	AGG TGG AAG GTT AGG AGG CA	ChIP qRT-PCR

Worksheet

All Events

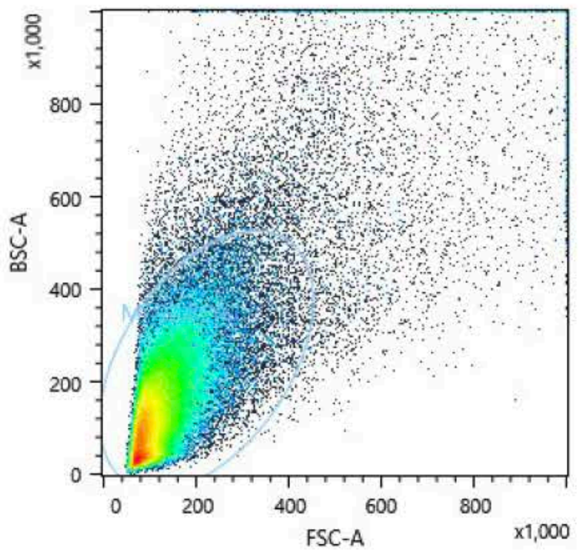

All Events

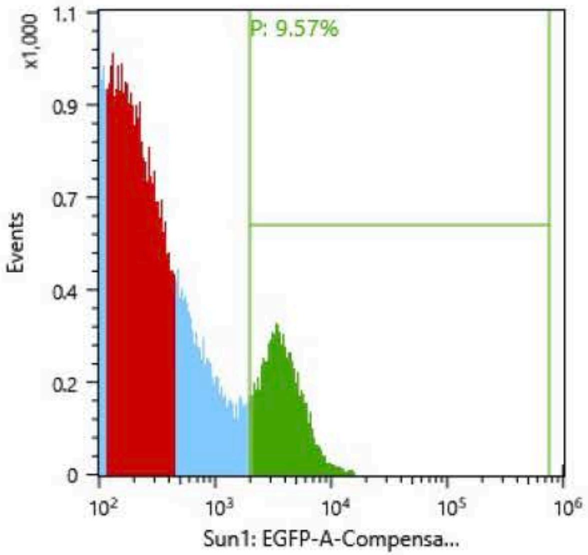

Gates and Statistics

Name	Events	%Parent	%Total
All Events	100,000	0.00%	100.00%
M	92,846	92.85%	92.85%
O	6,711	7.23%	6.71%
Q	28,641	30.85%	28.64%
P	9,572	9.57%	9.57%

M

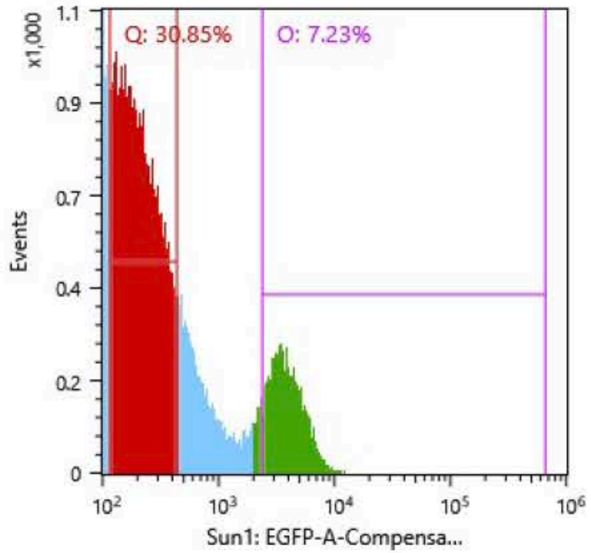

Supplement: Supplementary file 1 — Supplementary Information [file 41467_2023_42752_MOESM1_ESM.pdf]
