# Supplementary material for: Systemic and intrinsic functions of ATRX in glial cell fate and CNS myelination in male mice
Source: Nat Commun. 2023 Nov 4;14:7090. doi: 10.1038/s41467-023-42752-y (PMC10625541; doi:10.1038/s41467-023-42752-y)
Supplement: Supplementary file 2 — Reporting summary [file 41467_2023_42752_MOESM2_ESM.pdf]

nature portfolio

Corresponding author(s): Nathalie G. Berube

Last updated by author(s): September 26, 2023

Reporting Summary

Nature Portfolio wishes to improve the reproducibility of the work that we publish. This form provides structure for consistency and transparency in reporting. For further information on Nature Portfolio policies, see our Editorial Policies and the Editorial Policy Checklist.

Statistics

For all statistical analyses, confirm that the following items are present in the figure legend, table legend, main text, or Methods section.

n/a	Confirmed	
☐	☒	The exact sample size (*n*) for each experimental group/condition, given as a discrete number and unit of measurement
☐	☒	A statement on whether measurements were taken from distinct samples or whether the same sample was measured repeatedly
☐	☒	The statistical test(s) used AND whether they are one- or two-sided<br>*Only common tests should be described solely by name; describe more complex techniques in the Methods section.*
☒	☐	A description of all covariates tested
☒	☐	A description of any assumptions or corrections, such as tests of normality and adjustment for multiple comparisons
☐	☒	A full description of the statistical parameters including central tendency (e.g. means) or other basic estimates (e.g. regression coefficient) AND variation (e.g. standard deviation) or associated estimates of uncertainty (e.g. confidence intervals)
☐	☒	For null hypothesis testing, the test statistic (e.g. *F*, *t*, *r*) with confidence intervals, effect sizes, degrees of freedom and *P* value noted<br>*Give P values as exact values whenever suitable.*
☒	☐	For Bayesian analysis, information on the choice of priors and Markov chain Monte Carlo settings
☒	☐	For hierarchical and complex designs, identification of the appropriate level for tests and full reporting of outcomes
☒	☐	Estimates of effect sizes (e.g. Cohen's *d*, Pearson's *r*), indicating how they were calculated

Our web collection on statistics for biologists contains articles on many of the points above.

Software and code

Policy information about availability of computer code

Data collection

Openlab software (PerkinElmer Version 5.0, RRID:SCR_012158) was used for fluorescence image capture.

Data analysis

The paired-end reads in Fastq format were trimmed with Trim Galore (v.0.6.6), a wrapper tool around Cutadapt package, and were then aligned to the mouse genome (mm10) using Bowtie2(v.2.4.4) with the default parameters. SAMtools (v.1.12) was then used to create and sort BAM files from the aligned reads recorded in SAM format. The duplicated reads were marked with MarkDuplicates function from Picard (v.2.26.3). The mitochondrial DNA reads and blacklist regions of the genome were filtered out using Bedtools intersect (v.2.30.0).

MACS2 (v.2.2.7.1) broadPeak mode was used to call the peaks from the filtered BAM files with input control. Second, R package csaw (v.1.26.0) was used to count the reads in 300bp non-overlapping windows. Background noise was estimated by counting reads in 2000bp bins. We selected 300bp windows that have a signal higher than log2(3) above background. Windows were then merged if less than 100bp apart but did not extend above 5kb width. Rtracklayer (v.1.52.1) package was then used to export the filtered windows in a BED format.

findMotifsGenome.pl and annotatePeaks.pl functions from HOMER (v.4.10) were then used to find enriched DNA motifs in the peak lists and annotate the peaks in the genome, respectively. ATRX ChIP Bigwig track was generated using Deeptools2 bamCompare (v.3.5.2) with the parameter "—scaleFactorsMethod SES" (input control was used for normalization). plotHeatmap and plotProfile functions of Deeptools2 were also used to compare ATRX enrichment at peaks of publicly available ChIP-Seq datasets.

UCSC liftOver tool was used to convert peak coordinates from rn4 to mm10 genome assembly. The reads were aligned to rn4 genome assembly using Bowtie2 (v.2.4.4). SAMtools (v.1.12) was then used to generate and filter BAM files. Peaks were called as described for ATRX ChIP. UCSC liftOver was then used to convert peak coordinates from rn4 to mm10 genome assembly.

Wig files were converted to bigwig files using wigToBigWig from rtracklayer (v.1.52.1). Bigwig files were then converted to bedGraph files using UCSC bigWigToBedGraph tool. UCSC liftOver was then used to convert peak coordinates from mm8 to mm10 genome assembly.

Western blots were quantified using Fiji: an open-source platform for biological-image analysis. Cell counts were performed using Volocity (PerkinElmer Demo Version 6.0.1, RRID:SCR_002668). Image processing was performed using Volocity (PerkinElmer Demo Version 6.0.1, RRID:SCR_002668) and Adobe Photoshop. Fluorescence-activated nuclei sorting was analyzed using Sony SH800 Cell Sorter software. Statistical analysis was performed using GraphPad Prism6 software (6.05; GraphPad Software Inc.).

For manuscripts utilizing custom algorithms or software that are central to the research but not yet described in published literature, software must be made available to editors and reviewers. We strongly encourage code deposition in a community repository (e.g. GitHub). See the Nature Portfolio guidelines for submitting code & software for further information.

Data

Policy information about availability of data

All manuscripts must include a data availability statement. This statement should provide the following information, where applicable:

- Accession codes, unique identifiers, or web links for publicly available datasets
- A description of any restrictions on data availability
- For clinical datasets or third party data, please ensure that the statement adheres to our policy

The datasets for the AtrxFoxg1Cre RNA microarrays have been deposited in the National Centre for Biotechnology Information Gene Expression Omnibus Database under accession code GSE210863 (https://www.ncbi.nlm.nih.gov/geo/query/acc.cgi?acc=GSE210863), ATRX OPC nuclei RNA-seq and ATRX OPC ChIP-seq data can be accessed at NCBI Bioproject PRJNA866270 (https://www.ncbi.nlm.nih.gov/bioproject/PRJNA866270). Other datasets used in this study can be accessed at the NCBI GEO Database under the accession codes GSE116601 (ChIP-seq OPC CHD7 and CHD8), GSE42454 (ChIP-seq OPC Olig2 and H3K27Ac), GSE76412 (ChIP-seq OPC HDAC3 and P300) and GSE69859 (ChIP-seq NSC, Sox2, Brn-1 and Oct6). Source data are provided with this paper.

Research involving human participants, their data, or biological material

Policy information about studies with human participants or human data. See also policy information about sex, gender (identity/presentation), and sexual orientation and race, ethnicity and racism.

Reporting on sex and gender	This study does not involve human participants
Reporting on race, ethnicity, or other socially relevant groupings	This study does not involve human participants
Population characteristics	This study does not involve human participants
Recruitment	This study does not involve human participants
Ethics oversight	This study does not involve human participants

Note that full information on the approval of the study protocol must also be provided in the manuscript.

Field-specific reporting

Please select the one below that is the best fit for your research. If you are not sure, read the appropriate sections before making your selection.

☒ Life sciences ☐ Behavioural & social sciences ☐ Ecological, evolutionary & environmental sciences

For a reference copy of the document with all sections, see nature.com/documents/nr-reporting-summary-flat.pdf

Life sciences study design

All studies must disclose on these points even when the disclosure is negative.

Sample size	Sample sizes were not determined using power analysis. To minimize the number of mice used, we performed analysis on three or more biological replicates which is typical in the field of cell and molecular biology.
Data exclusions	No data was excluded
Replication	All attempts at replication were successul
Randomization	AtrxFoxG1Cre mice were randomly assigned to receive either vehicle or thyroxine. Cages of experimental mice of different genotypes were randomly positioned in the animal room.

Blinding	The quantification of immunofluorescent images was done in a blinded manner. Data collection and analysis for other experiments was not blinded as the data is quantitiative and is not subjective.

Behavioural & social sciences study design

All studies must disclose on these points even when the disclosure is negative.

Study description	N/A
Research sample	N/A
Sampling strategy	N/A
Data collection	N/A
Timing	N/A
Data exclusions	N/A
Non-participation	N/A
Randomization	N/A

Ecological, evolutionary & environmental sciences study design

All studies must disclose on these points even when the disclosure is negative.

Study description	N/A
Research sample	N/A
Sampling strategy	N/A
Data collection	N/A
Timing and spatial scale	N/A
Data exclusions	N/A
Reproducibility	N/A
Randomization	N/A
Blinding	N/A

Did the study involve field work? ☐ Yes ☒ No

Reporting for specific materials, systems and methods

We require information from authors about some types of materials, experimental systems and methods used in many studies. Here, indicate whether each material, system or method listed is relevant to your study. If you are not sure if a list item applies to your research, read the appropriate section before selecting a response.

Materials & experimental systems

n/a	Involved in the study	
☐	☒	Antibodies
☒	☐	Eukaryotic cell lines
☒	☐	Palaeontology and archaeology
☐	☒	Animals and other organisms
☒	☐	Clinical data
☒	☐	Dual use research of concern
☒	☐	Plants

Methods

n/a	Involved in the study	
☐	☒	ChIP-seq
☐	☒	Flow cytometry
☒	☐	MRI-based neuroimaging

Antibodies

Antibodies used

The following primary antibodies were used: anti-MOG, rabbit polyclonal (1:200, Abcam Cat# ab32760, RRID:AB_2145529), anti-MBP, rat monoclonal (1:50, Abcam Cat# ab7349, RRID:AB_305869), anti-MAG, mouse monoclonal (1:3000, Abcam Cat# ab89780, RRID:AB_2042411, anti-ATRX, rabbit polyclonal (1:75, Santa Cruz Biotechnology Cat# sc-15408, RRID:AB_2061023), anti-Olig2, rabbit polyclonal (1:200, Millipore Cat# AB9610, RRID:AB_570666), anti-Olig2, mouse monoclonal, clone 211F1.1 (1:100, Sigma Cat# MABN50, RRID:AB_10807410) anti-S100β, rabbit polyclonal (1:200, Agilent Cat# Z0311, RRID:AB_10013383), anti-GFAP, rabbit polyclonal (1:200, Agilent Cat# Z0334, RRID:AB_10013382), anti-NG2, rabbit polyclonal (1:200, Millipore Cat# AB5320, RRID:AB_11213678), anti-PDGFRα, rabbit polyclonal (1:200 Abcam Cat# ab65258, RRID:AB_1141669), anti-APC, mouse monoclonal (Abcam Cat# ab16794, RRID:AB_443473, anti-Ki67, rabbit polyclonal (1:150 Abcam Cat# ab15580, RRID:AB_443209), anti-NFIA (1:100 Sigma-Aldrich Cat# HPA006111, RRID:AB_1854422) and anti-Sox10, rabbit polyclonal (1:100, Abcam Cat# ab155279, RRID:AB_2650603).

The secondary antibodies used were goat anti-rabbit-Alexa Fluor 594 (1:800, Thermo Fisher Scientific, A-11012, RRID:AB_2534079), goat anti-rabbit-Alexa Fluor 488 (1:800, Thermo Fisher Scientific Cat# A-11008, RRID:AB_143165), goat anti-mouse-Alexa Fluor 594 (1:800 Thermo Fisher Scientific Cat# A-21125, RRID:AB_2535767), goat anti-mouse-Alexa Fluor 488 (1:800, Thermo Fisher Scientific Cat# A-11001, RRID:AB_2534069), goat anti-rat-Alexa Fluor 488 (1:800, Thermo Fisher Scientific, A-11006, RRID:AB_2534074), goat anti-mouse-Alexa 647 (1:800, Thermo Fisher Scientific Cat# A-21235, RRID:AB 2535804), mouse anti-HRP (1:3,000, Santa Cruz Cat# sc-516102, RRID:AB_2687626), rabbit anti-HRP (1:5000, Jackson ImmunoResearch Cat# 111-036-003, RRID:AB_2337942) or rat anti-HRP (1:3,000, Santa Cruz Cat# sc-2006, RRID:AB_1125219).

Validation

RRID:AB_2145529: 38 Published Figures from 21 Publications. Manufacturer recommends: western blot, immunohistochemistry; RRID:AB_305869: Independent validation by the NYU Lagone was performed. 483 Published Figures from 236 Publications. Applications:ELISA, IHC-FoFr, IHC-Fr, IHC-P, RIA, WB; Immunohistochemistry; Chromatography; Immunohistochemistry - fixed; Immunofluorescence; ELISA; Immunohistochemistry - frozen; Radioimmunoassay; RRID:AB_2042411. 69 Published Figures from 47 Publications. Manufacturer recommends: Immunohistochemistry, ELISA, Western Blot; RRID:AB_2061023: 151 Published Figures from 62 Publications. Manufacturer recommends: ELISA; Immunocytochemistry; Immunofluorescence; Immunohistochemistry; Immunoprecipitation; Western Blot; Immunoprecipitation; ELISA; RRID:AB_570666: Independent validation by the NYU Lagone was performed. 1100 Published Figures from 616 Publications. Applications: IC, IH, IH-P, IP, WB; RRID:AB_10807410: Published Figures from 145 Publications. Applications: WB, ICC, IHC, IP; RRID:AB_10013383: Independent validation by the NYU Lagone was performed. 547 Published Figures from 351 Publications. Manufacturer recommends: ELISA; Immunocytochemistry; Immunofluorescence; RRID:AB_10013382: Independent validation by the NYU Lagone was performed. 4300 Published Figures from 2600 Publications. Manufacturer recommends: Immunocytochemistry; RRID:AB_11213678: 9 Published Figures from 6 Publications. Manufacturer recommends: Immunocytochemistry; Immunoprecipitation; Immunohistochemistry; Western Blot; ELISA; RRID:AB_1141669: 15 Published Figures, from 8 Publications. Manufacturer recommends: Western Blot; Immunohistochemistry; RRID:AB_443473: 19 Published Figures from 16 Publications. Manufacturer recommends: Immunocytochemistry; Immunohistochemistry; Immunocytochemistry/Immunofluorescence; RRID:AB_443209: Independent validation by the NYU Lagone was performed. 2500 Published Figures from 1700 Publications. Manufacturer recommends: Immunocytochemistry; Immunohistochemistry - fixed; Immunohistochemistry - frozen; Flow Cytometry; Immunofluorescence; Immunohistochemistry; Western Blot; RRID:AB_1854422: 18 Published Figures from 11 Publications. Manufacturer recommends: Immunohistochemistry; Western Blot; RRID:AB_2650603: 107 Published Figures from 55 Publications. Manufacturer recommends: Immunohistochemistry; Western Blot; RRID:AB_2534079: 40 Published Figures, 2592 References. Applications: ICC/IF, Flow; RRID:AB_143165: 40 Published Figures, 8449 References. Applications: ICC/IF, Flow; RRID:AB_2535767: 3 Published Figures, 243 References. Applications: ICC/IF, Flow; RRID:AB_2534069: 40 Published Figures, 7384 References. Applications: ICC/IF, Flow; RRID:AB_2534074: 40 Published Figures, 1951 References. Applications: ICC/IF, Flow; RRID:AB 2535804: 12 Published Figures, 1414 References. Applications: ICC/IF, Flow; RRID:AB_2687626: 1502 References. Applications: Western blotting; RRID:AB_2337942: 66 References. Applications: Western blotting; RRID:AB_1125219: 271 references. Applications: Western blotting.

Animals and other research organisms

Policy information about studies involving animals; ARRIVE guidelines recommended for reporting animal research, and Sex and Gender in Research

Laboratory animals

Mus musculus (mice) were housed at a temperature of 20-26°C and humidity between 40-60%, exposed to 12-hour light/12-hour dark cycles and fed water and regular chow ad libitum. Foxg1promoter (129(Cg)-Foxg1tm1(cre)Skm/J, RRID:IMSR_JAX:004337). Experiments were performed at postnatal day (P)14 (thyroxine injected), P17 (microarray) or P20; Neurod6tm1(cre)Kan, MGI:2668659. Experiments were performed at P20; CBA;B6-Tg(Sox10-icre/ERT2)388Wdr, MGI:5634390, RRID:IMSR JAX:02765. Experiments were performed at P0.5, P3 (cultures), P35 (optic tract) or P20; B6;129-Gt(ROSA)26Sortm5(CAG-Sun1/sfGFP)Nat/J, MGI:5614796, RRID: IMSR_JAX:021039. Mice were bred into Sox10Cre line; B6.Cg-Gt(ROSA)26Sortm14(CAG-tdTomato)Hze/J, MGI:3809524, RRID:IMSR_JAX:007914. Mice were bred into Sox10Cre line.

Wild animals

The study did not involve wild animals.

Reporting on sex

This study focuses on ATRX, which is X-linked. Female carriers display highly skewed X chromosome inactivation toward the mutant allele and are usually phenotypically normal. Therefore, this study was performed only on male mice.

Field-collected samples

The study did not involve samples collected from the field

Ethics oversight

All procedures involving animals were conducted in accordance with the regulations of the Animals for Research Act of the Province of All procedures involving animals were conducted in accordance with the regulations of the Animals for Research Act of the province of Ontario, Canada and approved by the University of Western Ontario Animal Care and Use Committee (AUP 2017-048, 2021-062, 2021-049).

Note that full information on the approval of the study protocol must also be provided in the manuscript.

Plants

Seed stocks	N/A
Novel plant genotypes	N/A
Authentication	N/A

ChIP-seq

Data deposition

☒ Confirm that both raw and final processed data have been deposited in a public database such as GEO.

☒ Confirm that you have deposited or provided access to graph files (e.g. BED files) for the called peaks.

Data access links<br>*May remain private before publication.*	NCBI Bioproject PRJNA866270- https://dataview.ncbi.nlm.nih.gov/object/PRJNA866270?reviewer=2hmltrmblm1oofavpi9ujsij7b
Files in database submission	PRJNA866270- Oligodendocytes CTL RNA-seq 1,Oligodendocytes KO RNA-seq 1,Oligodendocytes KO RNA-seq 3,Oligodendocytes KO RNA-seq 2,ATRX ChIP-seq,ATRX Input, Oligodendocytes CTL RNA-seq 2, Oligodendocytes CTL RNA-seq 3
Genome browser session<br>(e.g. UCSC)	https://genome.ucsc.edu/cgi-bin/hgTracks?db=mm10&lastVirtModeType=default&lastVirtModeExtraState=&virtModeType=default&virtMode=0&nonVirtPosition=&position=chr1%3A3004600%2D3005200&hgsid=1464533075_zCC90fKMs6HbeZjS9wSnp7wTu1gV

Methodology

Replicates	1
Sequencing depth	25 million paired-end (150bp) reads were generated for every sample
Antibodies	anti-ATRX rabbit polyclonal antibody (Abcam Cat# ab97508, RRID:AB_10680289)
Peak calling parameters	macs2 callpeak -f BAMPE --broad -t opcAtrx27.bam -c opc2ipChip.bam -g 2.7e9 -n opcAtrx27_broad --outdir ../macs2_peaks
Data quality	The paired-end reads in Fastq format were trimmed using Trim Galore v.0.6.6. Picard MarkDuplicates v.2.26.3 was used to mark duplicated reads. FastQC v.0.11.9 was used to check for quality control of both raw and aligned reads. MultiQC v.1.12 was then used to summarize the output of aforementioned tools. For peak calling, broad mode in Macs2 v.2.2.7.1 was used with the default q-value cutoff of 0.01 resulting in 42820 peaks. 4836 of all the peaks had above 5-fold enrichment.
Software	Trim Galore v.0.6.6, Bowtie2 v.2.4.4, SAMtools v1.12, Picard MarkDuplicates v.2.26.3, Bedtools v.2.30.0, FastQC v.0.11.9, MultiQC v.1.12, Macs2 v.2.2.7.1, csaw v.1.26.0, Rtracklayer v.1.52.1, HOMER v.4.10, Deeptools2 v.3.5.2,

Flow Cytometry

Plots

Confirm that:

☒ The axis labels state the marker and fluorochrome used (e.g. CD4-FITC).

☒ The axis scales are clearly visible. Include numbers along axes only for bottom left plot of group (a 'group' is an analysis of identical markers).

☒ All plots are contour plots with outliers or pseudocolor plots.

☒ A numerical value for number of cells or percentage (with statistics) is provided.

Methodology

Sample preparation	Fluorescence-activated nuclei sorting (FANS) was used to purify Sun1-GFP+ OPC nuclei from brain tissue. Control or AtrxSox10Cre forebrain tissue was homogenized in 500 uL homogenization buffer (20 mM Tricine KOH, 25 mM MgCl2, 250 mM sucrose, 1 mM DTT, 0.15 mM spermine, 0.5 mM spermidine, 0.1% IGEPAL-630, 1x protease inhibitor cocktail (Millipore Sigma Cat# 11873580001), 1 uL/mL RNase inhibitor (Thermo Fisher Scientific Cat# 10777019). Samples were diluted to 7.5 mL with homogenization buffer and filtered through a 40 µm strainer. Filtered samples were layered on top of 7.5 mL cushion buffer consisting of 0.5 mM MgCl2, 0.88 M sucrose, 0.5 mM DTT, 1x protease Inhibitor cocktail (Millipore Sigma Cat# 11873580001), 1 uL/mL RNase inhibitor (Thermo Fisher Scientific Cat# 10777019) and centrifuged at 2800 g for 20 mins at 4°

	C. Nuclei were collected as a pellet, incubated for 10 min in 500 uL 4% FBS, 0.15 mM spermine, 0.5 mM spermidine, 1x protease inhibitor cocktail (Millipore Sigma Cat# 11873580001) and 1 uL/mL RNase inhibitor (Thermo Fisher Scientific Cat# 10777019) in PBS and resuspended by gentle pipetting. Samples were filtered through a 20 µm strainer (pluriStrainer 43-10020-60) before sorting with a 100 µm nozzle, and the following gains for the sensors: FSC:14, BSC: 35%, FL1(EGFP):38%.
Instrument	Nuclei were sorted using a Sony SH800 Cell Sorter and Sun1GFP+ nuclei were collected.
Software	H800S Cell Sorter software
Cell population abundance	From the total sorted nuclei, OPC nuclei with GFP were around 7-8. GFP+ purity of sorted nuclei was confirmed under fluorescent microscope and it was ~80% pure population.
Gating strategy	The gatings were optimized using GFP- samples, to determine the background levels of signal.

☒ Tick this box to confirm that a figure exemplifying the gating strategy is provided in the Supplementary Information.
